# Whole-Genome Sequencing of Shiga Toxin-Producing *Escherichia coli* for Characterization and Outbreak Investigation

**DOI:** 10.3390/microorganisms11051298

**Published:** 2023-05-16

**Authors:** Heather M. Blankenship, Stephen E. Dietrich, Elizabeth Burgess, Jason Wholehan, Marty Soehnlen, Shannon D. Manning

**Affiliations:** 1 Bureau of Laboratories, Michigan Department of Health and Human Services, Lansing, MI 48824, USA; blankenshiph@michigan.gov (H.M.B.);; 2 Department of Microbiology and Molecular Genetics, Michigan State University, East Lansing, MI 48824, USA

**Keywords:** Shiga toxin, *Escherichia coli*, outbreak, genome sequencing, phylogenetics

## Abstract

Shiga toxin-producing *Escherichia coli* (STEC) causes high frequencies of foodborne infections worldwide and has been linked to numerous outbreaks each year. Pulsed-field gel electrophoresis (PFGE) has been the gold standard for surveillance until the recent transition to whole-genome sequencing (WGS). To further understand the genetic diversity and relatedness of outbreak isolates, a retrospective analysis of 510 clinical STEC isolates was conducted. Among the 34 STEC serogroups represented, most (59.6%) belonged to the predominant six non-O157 serogroups. Core genome single nucleotide polymorphism (SNP) analysis differentiated clusters of isolates with similar PFGE patterns and multilocus sequence types (STs). One serogroup O26 outbreak strain and another non-typeable (NT) strain, for instance, were identical by PFGE and clustered together by MLST; however, both were distantly related in the SNP analysis. In contrast, six outbreak-associated serogroup O5 strains clustered with five ST-175 serogroup O5 isolates, which were not part of the same outbreak as determined by PFGE. The use of high-quality SNP analyses enhanced the discrimination of these O5 outbreak strains into a single cluster. In all, this study demonstrates how public health laboratories can more rapidly use WGS and phylogenetics to identify related strains during outbreak investigations while simultaneously uncovering important genetic attributes that can inform treatment practices.

## 1. Introduction

Shiga toxin-producing *Escherichia coli* (STEC) is a Gram-negative foodborne pathogen that was estimated to cause ~265,000 infections, 3600 hospitalizations, and 30 deaths in the U.S. each year [1]. Most patients with STEC infections develop diarrhea and abdominal pain, though hemolytic uremic syndrome (HUS) and kidney failure can occur, especially in young children [1,2]. STEC was first identified in the early 1980s and has been linked to numerous outbreaks from a wide range of sources, including beef and dairy products and fresh produce [3,4]. Since its identification, STEC O157 has been responsible for most infections; however, the number of cases with non-O157 STEC infections has increased gradually over time [5,6]. This increase is partly due to enhanced surveillance and detection methods targeting non-O157 serogroups [5,6,7].

Foodborne transmission was estimated to account for 85% of O157 STEC infections annually [8] as well as 84% of the non-O157 outbreaks that occurred prior to 2010 [4]. Enhancing the ability to accurately track infection sources is critical for disease prevention efforts. Until recently, the Centers for Disease Control and Prevention (CDC) used pulsed-field gel electrophoresis (PFGE) as the gold standard for STEC surveillance [9]. Although PFGE standardization allows for the comparison of banding patterns across public health laboratories [10], the method is time- and labor-intensive and lacks the discriminatory power to confirm that two strains are genetically identical [11]. PFGE also prevents a comprehensive evaluation of evolutionary relationships and lacks the ability to detect and characterize pathogen virulence traits without the use of pathogen-specific typing methods [11]. Hence, there is a critical need for alternative typing tools that can more efficiently and effectively discriminate between foodborne pathogens and confirm outbreak sources.

Increased use of whole-genome sequencing (WGS) analyses using Next-Generation Sequencing platforms has enhanced our understanding of pathogen diversity and was suggested to replace PFGE for classifying and characterizing foodborne pathogens [11]. Application of WGS, for instance, was instrumental in the characterization of the Shiga toxin-producing enteroaggregative *Escherichia coli* O104:H4 German outbreak strain [12], which was distinct from STEC O104 strains recovered previously [13,14]. Moreover, sequencing of additional O104:H4 strains linked to the same outbreak detected differences, with strains from cases in France and Germany differing by 19 single nucleotide polymorphisms (SNPs) [15]. This result contrasted the data generated by PFGE and rep-PCR, which found the strains to be indistinguishable [15].

In the clinical laboratory setting, the use of WGS enables an assessment of the genetic relatedness of strains and the identification of important genetic factors. Such factors include genes encoding resistance to antibiotics and virulence, as well as O- and H-antigen genes used for serotyping, which are critical for surveillance [16,17,18]. WGS also allows for the identification of pathogens that are evolving more rapidly through the detection of plasmids and mobile genetic elements that can be transferred between bacterial populations. Enhanced detection of these elements is due to the increased availability of public databases such as Virulence Finder [19] and SuperPhy [20] for STEC, which promote the rapid detection of genes that are prone to horizontal transfer [21]. Moreover, a wide range of methods are available for efficiently assembling and annotating bacterial genomes, thereby making the application of WGS analyses more user-friendly.

Prior studies have shown that WGS can identify and genotype pathogens more quickly and precisely than traditional methods while providing better resolution [22,23]. Application of WGS to foodborne pathogens such as *Listeria monocytogenes*, contributed to the identification of more outbreaks in one year when compared to the use of conventional methods [24]. The same study also demonstrated that more listeriosis outbreaks were solved, or linked to a source, since WGS had been implemented. As a result, the CDC has developed detailed WGS guidelines for public health laboratories to detect and characterize *L. monocytogenes*, STEC, and other foodborne pathogens such as *Shigella flexneri*, *Salmonella* spp., and *Campylobacter* spp. [25]. Such protocols are important for standardization and comparison across public health laboratories.

Herein, we have completed a retrospective WGS analysis of STEC strains recovered from patients between 2015 and 2018 for comparison to PFGE data, the prior gold standard method. This study has enabled a complete genomic assessment of STEC in circulation and provided confirmation of linkages with known outbreak strains. Indeed, comparing PFGE patterns to WGS-based phylogenies has promoted the identification of specific strains that should have been included in prior outbreaks. These findings demonstrate the importance of using similar bioinformatic approaches for outbreak investigations and source attribution studies in the future.

## 2. Materials and Methods

### 2.1. Bacterial Strains and NGS

The Michigan Department of Health and Human Services (MDHHS) recovered and sequenced 625 clinical isolates during 2015–2018 that were preliminarily classified as STEC or *Shigella*. Ethical review and approval were not required for this study in accordance with local legislation and institutional requirements. Isolates were grown overnight at 37 °C with aeration and prepped for sequencing using standard operating procedures established for PulseNet by the CDC (https://www.cdc.gov/pulsenet/pathogens/wgs.html (accessed on 1 July 2019). DNA was extracted with the Qiagen DNeasy Kit (Qiagen, Valencia, CA, USA), libraries were prepared using the Nextera XT kit (Illumina, San Diego, CA, USA), and sequencing was performed on the Illumina MiSeq platform (2 × 250 reads).

Prior to read processing and analysis, Kraken [26] was used to identify those isolates that were classified as *Shigella* for removal. Preprocessing of the reads was performed with TrimmomaticPE [27] to remove adapters as well as reads with a phred quality score lower than 20 (Q20) and lengths less than 100 nucleotides. Quality control checking of the sequences was performed with FastQC v 0.11.8 [28], and de novo assemblies were performed with Spades v 3.10.1 using kmers 21, 33, 55, 77, 99, and 127 as described previously [29]. Default parameters were used for assembly, and error correction was applied to reduce the number of mismatches.

### 2.2. Pulsed-Field Gel Electrophoresis (PFGE)

PFGE was performed on clinical isolates as part of the standard operating procedures outlined by the CDC for STEC via the PulseNet national surveillance system [10]. PFGE patterns were analyzed using BioNumerics 7.5 (Applied Maths, Austin, TX, USA), and outbreak codes were assigned by the CDC if the PFGE patterns matched those of other isolates found in the database [25].

### 2.3. Bioinformatic Analyses

Genes that have been linked to virulence or are useful for *in silico* serotyping (e.g., *wzy/wzx* and *fliC* encoding the O- and H-antigen, respectively) were extracted from the genomes with ABRicate v.2 (https://github.com/tseeman/abricate) (accessed on 15 September 2019) using databases downloaded from the Center for Genomic Epidemiology (http://www.genomicepidemiology.org/) (accessed on 15 September 2019). Sequences were extracted from the National Center for Biotechnology Information (NCBI) database for the following STEC virulence gene alleles: *stx1* (a–d) and *stx2* (a–g) encoding the Shiga toxins, 14 *eae* (intimin) alleles; and six *ehxA* (enterohemolysin) subtypes as described [17]. Seven gene sequences were also extracted for multilocus sequence typing (MLST) using in-house Python (v 3.5) scripts developed with the Basic Local Alignment Search Tool (BLAST)+ platform available through the NCBI (accessed on 15 September 2019) [30]. Sequence types (STs) were assigned using the Whittam scheme (EcMLST v. 1.2), available through the STEC Center at Michigan State University (http://www.shigatox.net) (accessed on 30 September 2019) [31]. MLST alleles were concatenated and aligned with CLUSTALW, and a similarity tree was generated using the Neighbor-joining algorithm with 1000 bootstrap replications in MEGA X [32]; *E. coli* K12 [33] was included for reference.

Preliminary core genome single nucleotide polymorphism (cgSNP) analysis was performed by annotating all genomes and aligning the shared genes with Parsnp using default parameters as described [34]. The cgSNP analysis was applied to strains comprising large clusters in the MLST tree that included known outbreak-associated strains. For this analysis, a genome within each cluster was used as the reference for cgSNP detection. To ensure that the same cgSNPs were identified and the analysis was not skewed by a single genome, multiple genomes were evaluated as reference genomes for most clusters. This approach also ensured that we did not miss any virulence regions that may have been specific to the cluster of isolates examined. After the cgSNPs were extracted from each set of genomes, they were input into FastTree 2 to infer approximate maximum-likelihood phylogenies based on the SNP nucleotide alignments [35]. Clade confidence was estimated using an approximate likelihood-ratio test (SH-like) [36], with the branch lengths representing substitutions per core-genome site.

For strains that grouped together with known outbreak strains in the cgSNP phylogeny, a high-quality SNP (hqSNP) analysis was performed on all strains within each cluster. hqSNP trees were generated from raw reads using Lyve-SET with RAxML to infer the maximum-likelihood phylogenies from the SNP alignments using parameters described for STEC [37], as we have done previously [38]. These parameters required each SNP to have a ≥95% identity to the reference sequence and at least a 20-read depth with two forward and two reverse reads in the region containing each SNP [37]. TreeGraph 2 [39] and FigTree (http://tree.bio.ed.ac.uk/software/figtree) (accessed on 15 September 2019) were used to visualize the dendrograms. An overview of the bioinformatic pipelines is described in Figure S1.

## 3. Results

### 3.1. Isolate Identification and Changing Serogroup Distributions over the Surveillance Period

**Figure 1:**
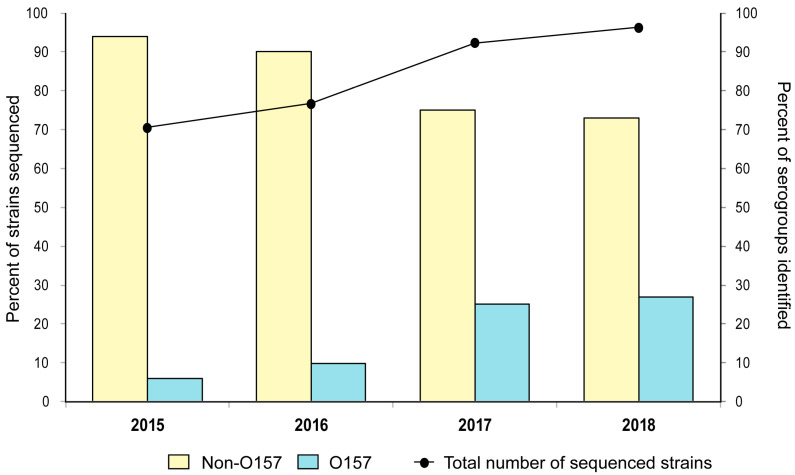
Frequency of Shiga toxin-producing *Escherichia coli* (STEC) isolates (*n* = 510) that were subjected to whole-genome sequencing in Michigan per year (black line) and the overall frequency (%) of non-O157 and O157 serogroups identified for each of the four years.

WGS for STEC was introduced at the MDHHS in 2015 to enhance surveillance activities and enable more thorough outbreak investigations. A total of 625 probable *Shigella* and diarrheagenic *E. coli* isolates were sequenced and given PNUSAE identifiers per the CDC protocol. WGS classified 97 (15.5%) of these isolates as *Shigella* spp., while 18 (2.9%) lacked *stx* genes; all 115 of these isolates were excluded from the study, leaving a total of 510 STEC isolates for the final analysis. Since its introduction, use of WGS for STEC isolates has increased from 70.6% to 96.3% of all isolates recovered in Michigan between 2015 and 2018, respectively (Figure 1). Despite the predominance of non-O157 serogroups identified each year, the number of sequenced non-O157 strains decreased from 94.0% to 73.1% over the time period. The number of sequenced O157 strains, however, increased sharply from 6.0% to 26.9%, which likely contributed to the overall increase observed. A subset of 87 isolates was not sequenced due to potential isolate duplication or low prioritization since PFGE remained the gold standard until 2019. Among these 87 isolates without sequencing data, most were recovered in 2015 (*n* = 35) or 2016 (*n* = 34) and were classified as O157 (*n* = 60; 68.9%).

Over the four-year period, 34 typeable serogroups were identified, with serogroup O103 predominating (*n* = 111; 21.8%) among the 510 isolates (Table S1). Serogroups O157 (*n* = 98; 19.2%), O45 (*n* = 61; 12.0%), O26 (*n* = 51; 10.0%), O111 (*n* = 47; 9.2%), and O121 (*n* = 28; 5.5%) were also common. Isolates representing these five non-O157 serogroups comprised 58.4% (*n* = 298) of the 510 isolates examined. Other serogroups were also detected and include: O5 (*n* = 15), O71 (*n* = 17), O123 (*n* = 13), and O151 (*n* = 10). In all, nine isolates could not be serogrouped due to incomplete or missing *wzx/wzy* genes; all nine isolates were classified as non-typeable (NT). Another isolate was classified as lacking the H-antigen [H-] due to incomplete sequencing of *fliC*.

### 3.2. Strain Characterization and Discrimination by PFGE and MLST

Among the 510 strains, PFGE detected 352 unique PFGE patterns following digestion with *Xba*I, indicating a high level of genetic variation among the clinical STEC strains in circulation. Comparatively, MLST classified 509 of these strains into 46 distinct STs with 60 serogroup/ST combinations; one O103:H2 isolate had incomplete sequences for all seven genes, and the ST could not be determined. In all, the most common genotype was ST-119, which mostly comprised serotype O103:H2 (*n* = 108; 21.2%) strains, while ST-66 strains representing serotype O157:H7 (*n* = 96; 18.9%) were also common. All O157 strains were classified as ST-66 except for two that were designated as novel STs (ST-1216).

**Figure 2:**
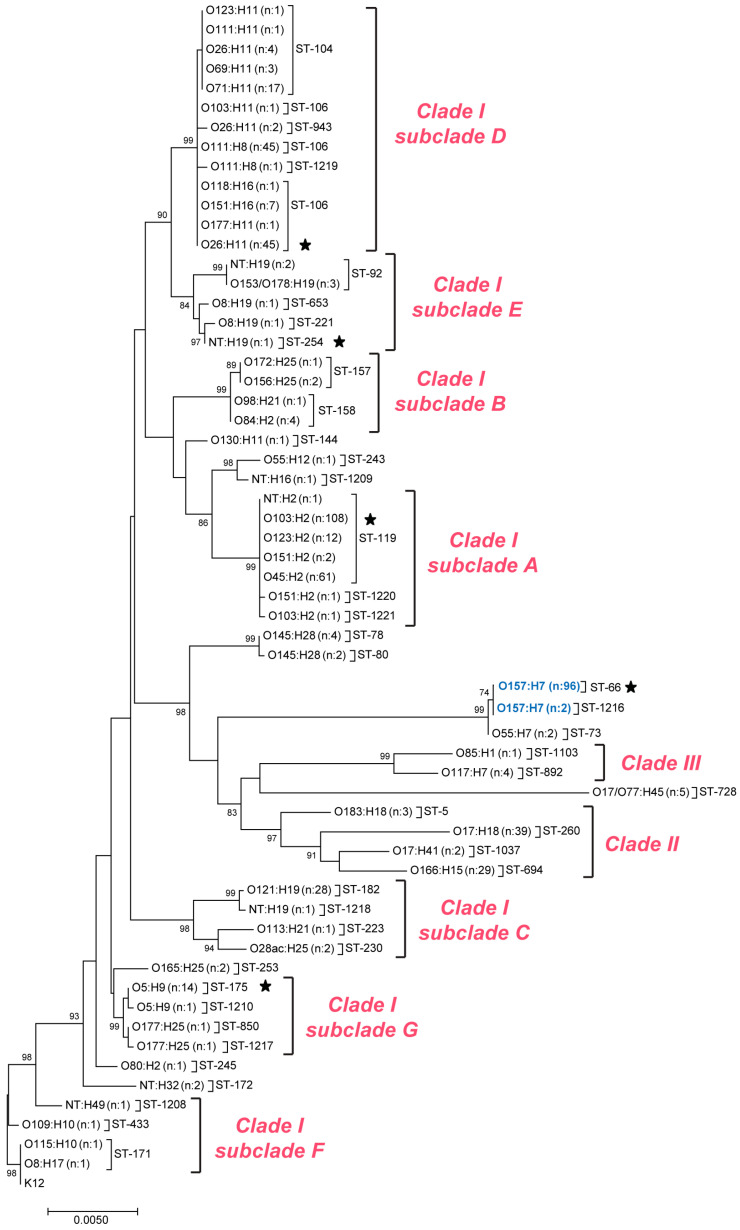
Neighbor-joining tree based on seven multilocus sequence typing loci for 509 Shiga toxin-producing *Escherichia coli* (STEC) isolates constructed with 1000 bootstrap replicates. Sequence types (STs) are indicated after each serotype along with the number (n) of strains examined, and bootstrap percentages (>80%) are shown at the nodes. Serogroup O157 strains are noted in blue font. Black stars indicate the STs and serogroups containing outbreak-associated isolates that were previously identified using pulsed-field gel electrophoresis.

Next, a Neighbor-joining tree was constructed using the seven MLST loci to determine the similarity between the clinical isolates and outbreak-associated isolates (Figure 2). The strains were grouped together into the same clades and subclades defined previously for an overlapping subset of non-O157 STEC strains [17]. Intriguingly, the O157 ST-1216 strain clustered with the 96 ST-66 O157:H7 strains within the MLST tree, along with the two ST-73 O55:H7 strains. Most of the clusters in the tree, however, comprised more than 15 strains, while five contained at least one outbreak-associated strain as determined by PFGE. Twenty-six outbreak strains representing ST-106 (O26:H11; *n* = 1), ST-119 (O103:H2; *n* = 2), ST-254 (NT:H19; *n* = 1), ST-66 (O157:H7; *n* = 16), and ST-175 (O5:H9; *n* = 6) were selected for the subsequent core genome analyses.

### 3.3. Core Genome (cg) SNP and High Quality (hq) SNP Analyses Can Retrospectively Detect Misclassified Outbreak Strains

A cgSNP analysis was first applied to two *stx1a*-positive outbreak strains with matching PFGE patterns and outbreak codes despite having different O-antigens (serogroups) and H-antigens. One strain was classified as O26:H11, ST-106 (PNUSAE001586), whereas the other was a NT:H19 strain belonging to ST-254 (PNUSAE001592). Both strains were found on different branches in the MLST tree within subclades D and E, respectively, though these subclades grouped together with 90% bootstrapping (Figure 2). To better differentiate these closely related subclades, a cgSNP analysis was performed on all 137 strains. In this analysis, four distinct clusters were identified, though the two outbreak strains were located in different clusters (Figure S2). It is therefore likely that these strains are not genetically identical and were misclassified as being part of the same outbreak; inclusion in the outbreak was previously determined based on the original PFGE result and similar case diagnosis dates. Additional support for misclassification was provided by the remaining 135 strains included in the cgSNP phylogeny, which were not previously considered to be part of the same outbreak since they had different PFGE patterns. Because of these findings, a subsequent hqSNP analysis of the two outbreak strains was deemed unnecessary, and instead, both genomes were compared. A total of 25,037 SNPs differed between the O26 and NT outbreak strains, which also possessed distinct *eae* and *ehx* alleles, providing even more support for the misclassification of one or the other outbreak strain.

**Figure 3:**
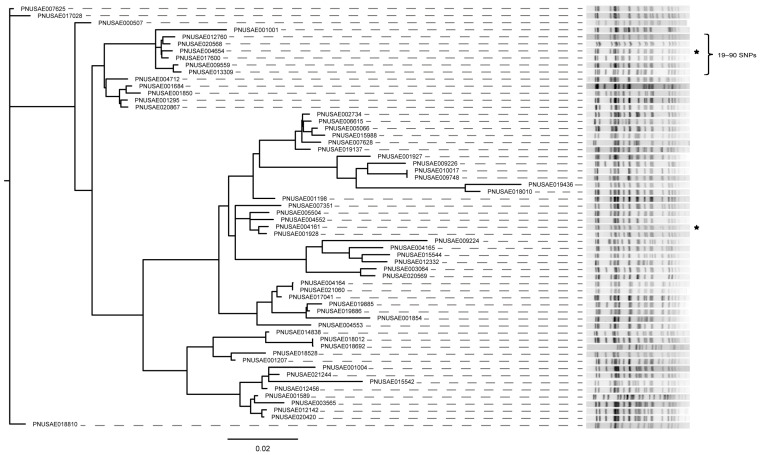
Dendrogram based on high-quality (hq) SNP analysis of 60 sequence type (ST)-119 Shiga toxin-producing *Escherichia coli* (STEC) strains that clustered with known outbreak strains in the cgSNP analysis. PFGE patterns (*Xba*I) are shown for all STEC isolates included in the hqSNP analysis and outbreak-associated isolates are denoted with stars. Brackets indicate the number of SNP differences between the specified group of strains.

Core genome analyses were also performed on the 186 strains within subclade A of Clade I, which grouped together with 99% bootstrapping in the MLST tree (Figure 2); multiple serotypes were represented, including O103:H2 (*n* = 108) and O45:H2 (*n* = 61). Although most of these strains belonged to ST-119, strains representing STs 1220 and 1221 were also included. Despite having similar collection dates as well as identical PFGE patterns, outbreak codes, and *eae* and *ehx* alleles, the cgSNP analysis indicated the two *stx1a*-positive O103:H2 outbreak-associated strains, PNUSAE004161 and PNUSAE004654, were distinct (Figure S3). Therefore, these two strains should not have been included as part of the same outbreak. To compare the genetic relatedness of the 60 ST-119 strains that clustered with the two outbreak strains in the cgSNP analysis, a hqSNP analysis was performed. In this analysis, the two outbreak strains comprised distinct clusters in the hqSNP phylogeny with up to 120 SNP differences (Figure 3), providing evidence of dissimilarity and misclassification. Interestingly, the hqSNP analysis also demonstrated that outbreak strain PNUSAE004654 was part of a distinct cluster of non-outbreak strains with similar PFGE profiles differing by only 19–90 SNPs.

### 3.4. cgSNP and hqSNP Analyses Can Accurately Differentiate Outbreak Strains

#### 3.4.1. STEC O157:H7

For the 98 STEC O157:H7 strains recovered between 2015 and 2018, 16 outbreak-associated strains were identified by PFGE. These strains were all classified as ST-66 but belonged to three different outbreaks. Since 82 additional ST-66 STEC O157:H7 strains were recovered during an overlapping time period, all 98 genomes were selected for inclusion in the cgSNP analysis. Unlike the MLST-based tree, the ST-66 strains could be differentiated into multiple clusters (Figure S4). The six strains from outbreak 1 (ST-66-O1) grouped together into a single cluster along with nine non-outbreak-associated strains. Although three of these nine non-outbreak strains had the same *XbaI* PFGE pattern as the outbreak strains, none were assigned the same outbreak code by the CDC. On the other hand, outbreaks 2 (ST-66-O2, *n* = 3 strains) and 3 (ST-66-O3, *n* = 6 strains), which were distinct by PFGE, were grouped together within the same cgSNP cluster along with four non-outbreak strains. Within this cluster, the six ST-66-O3 outbreak strains were most closely related to each other, while the three ST-66-O2 outbreak strains were located on two different branches of the phylogeny. Such findings highlight the need to apply more discriminatory genomic approaches to better differentiate closely related strains.

**Figure 4:**
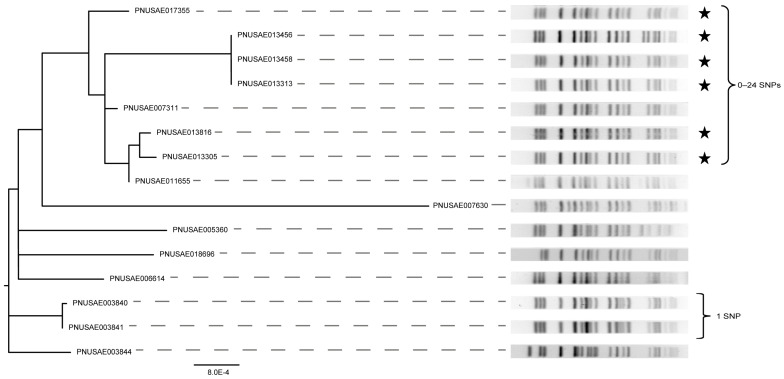
High-quality (hq) SNP phylogeny and XbaI PFGE patterns for 15 isolates of STEC O157:H7 linked to an outbreak. The six outbreak isolates (ST-66-01, black stars) were compared to nine related O157:H7 isolates, as determined by the cgSNP analysis. Brackets show the number of SNP differences between the specified cluster.

Indeed, hqSNP analysis was applied to the subset of O157 ST-66 strains comprising the two different clusters containing the outbreak strains in the cgSNP phylogeny. The first analysis included the six outbreak 1 (ST-66-O1) strains plus nine other strains that were part of the same cgSNP cluster. Overlaying the PFGE data onto the hqSNP phylogeny showed that the banding patterns were highly similar for all of the strains in the cgSNP cluster (Figure 4). The six ST-66-O1 outbreak strains had the same *eae* and *ehx* alleles, yet four of the strains had both the *stx2a* and *stx2c* genes as opposed to *stx2c* alone. The six strains also had identical banding patterns, though a shift in a single band was observed in outbreak strain PNUSAE013456. Importantly, all six outbreak strains and one non-outbreak strain differed by only 0–24 SNPs. Based on this level of similarity and the banding pattern, the non-outbreak strain PNUSAE007311 could have been classified as part of the original outbreak if the epidemiological data supported inclusion. However, it was isolated a year earlier than the rest of the outbreak strains and was therefore not classified as part of the same outbreak.

**Figure 5:**
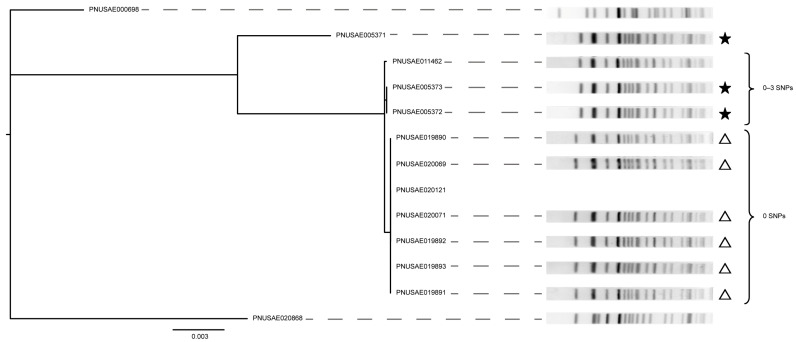
High-quality (hq) SNP phylogeny of 13 STEC O157:H7 isolates recovered from two outbreaks. Isolates from outbreaks ST-66-O2 (colored stars) and ST-66-O3 (open triangles) were included in the analysis as well as four non-outbreak isolates that clustered together in the cgSNP phylogeny. *Xba*I PFGE patterns are indicated for all but one isolate with missing data, while the brackets denote the number of SNP differences between the specified group of strains.

Given the high degree of relatedness between the nine ST-66 O157:H7 strains from outbreaks 2 (ST-66-O2) and 3 (ST-66-O3) and four non-outbreak strains in the cgSNP analysis (Figure S4), a hqSNP phylogeny was also constructed to enhance differentiation. Notably, strains from both outbreaks clustered together and differed by only 0–3 SNPs (Figure 5). All nine outbreak strains possessed *stx2a* and had identical *eae* and *ehx* alleles. Similar banding patterns were also observed for the nine outbreak strains, though strains belonging to the ST-66-O2 outbreak had a slight shift in the first band relative to strains from the ST-66-O3 outbreak. Outbreak strain PNUSAE005371 differed by additional SNPs but was recovered during the same time frame as the other two ST-66-O2 outbreak strains. Although the non-outbreak strain PNUSAE011462 was more similar to the ST-66-O2 outbreak strains with 0–3 SNP differences, it was recovered one year after the ST-66-O2 outbreak strains were detected and a year prior to the ST-66-O3 outbreak strains. The two most distantly related non-outbreak strains, PNUSAE000698 and PNUSAE020868, had similar PFGE patterns but failed to cluster with the known outbreak strains. Together, these findings indicate that the two O157 outbreaks were caused by the same strain. Since a year had passed between the two outbreaks, they were likely misclassified as distinct outbreaks; a continuous source was not investigated. In addition, two strains with missing and slightly distinct PFGE patterns were misclassified as non-outbreak strains. Hence, the use of WGS would have increased discriminatory power by identifying additional cases and linking all cases from both outbreak investigations together.

#### 3.4.2. STEC O5:H9 and O177:H25

WGS was also applied to the 17 closely related STEC strains belonging to Clade I, subclade G, that were grouped together with 99% bootstrapping in the MLST tree (Figure 2). These strains comprised serotypes O5:H9 (*n* = 15) and O177:H25 (*n* = 2) and represented four STs; ST-175 predominated among the O5:H9 strains, though one was classified as ST-1210. Importantly, a cgSNP analysis demonstrated that all strains in subclade G were highly related despite having different serotypes and STs. In the cgSNP phylogeny, the six ST-175 O5:H9 outbreak-associated strains were grouped together with a set of nine non-outbreak strains in the phylogeny, whereas two strains were more distantly related (Figure S5).

**Figure 6:**
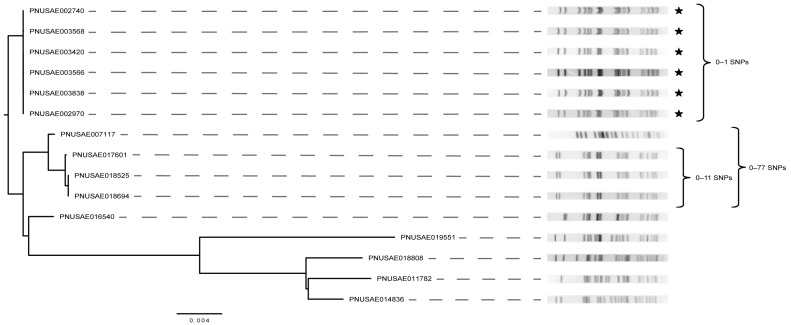
Phylogeny constructed using high-quality (hq) SNPs extracted from six STEC O5:H9 outbreak strain genomes, which are indicated with black stars. For comparison, nine other strains identified to be closely related in the core genome (cg) SNP analysis (Figure S4) were included. PFGE patterns were derived following digestion with *Xba*I; brackets indicate the number of SNP differences between a group of strains.

To better define the relatedness of the 15 strains comprising the primary cluster identified in the cgSNP phylogeny, a hqSNP analysis was performed. The six ST-175 O5:H9 outbreak strains clustered together in one distinct clade with only 0–1 SNP difference(s) (Figure 6). Since these six outbreak strains were also identical by PFGE, this analysis confirms that these outbreak strains were properly classified. Unlike the cgSNP phylogeny, the remaining nine strains were found on different branches in the hqSNP phylogeny, confirming that they are genetically distinct. Three ST-175 strains with a slightly different *Xba*I PFGE pattern relative to the outbreak strains clustered together on one branch and differed by 0–11 SNPs. All three strains had identical *stx*, *eae,* and *ehx* alleles and were recovered within a month of each other, though no outbreak code was assigned. Despite a similar allele profile and placement within the cgSNP phylogeny, however, strain PNUSAE007117 had a drastically different PFGE pattern but was found to differ from the three strains by only 0–77 SNPs in the hqSNP analysis. Upon further investigation, this strain was previously misclassified as O157, and hence, the use of WGS and the subsequent phylogenetic analyses would have more accurately classified the strain as being highly related to the three other O5:H9 strains. In this scenario, an outbreak investigation may have been warranted even though strain PNUSAE007117 was identified a year prior to the remaining three strains.

## 4. Discussion

The introduction of WGS into public health laboratories across the United States has improved surveillance efforts and enhanced our ability to detect enteric pathogens that may be epidemiologically linked or from a specific food source. Although the use of PFGE as the gold standard for surveillance promoted standardization across laboratories and enhanced national surveillance efforts [10,40], it lacks discriminatory power and prevents the application of phylogenetics [10,23,41,42]. Comparatively, WGS allows for a complete genomic analysis to be performed while replacing traditional microbiological methods with a shorter turnaround time [43,44,45,46]. WGS is also useful for typing strains that were previously unable to be typed due to the presence of novel serogroups, antigen cross-reactivity, or the unavailability of antibodies for a given serogroup [47,48]. While library preparation and sequencing methodology have been standardized by the CDC for public health laboratories, the analysis of sequencing data has been limited to laboratories with skilled bioinformaticians on staff or standard pipelines in place. With the switch from PFGE to WGS, the CDC has been analyzing all WGS data for national outbreaks until BioNumerics becomes fully functional and validated. Meanwhile, PFGE has been used simultaneously to prevent a lapse in surveillance activities.

Since WGS is still relatively new to public health laboratories, the reported molecular traits and serogroup distributions may not be representative of the true frequencies within a state or region. The prioritization and identification of certain serogroups may differ from the original implementation dates to the present. The recent sequencing trends for non-O157 and O157 STEC differ from what has been reported by the CDC through FoodNet, with a decrease in STEC O157 nationally but an increase in Michigan [5,6,49]. This discrepancy may be due to fewer O157 isolates getting sequenced in the first two years, as priority has been given to suspected outbreak strains and non-O157 serogroups. During 2015 and 2016, multiple non-O157 outbreaks occurred in Michigan and elsewhere. These included the 2016 O5:H9 (ST-175) outbreak linked to contaminated cheese examined herein and a 2016 multistate O26/O121 outbreak associated with contaminated flour [50,51].

Through this retrospective analysis of 509 STEC genomes from Michigan patients, we were able to compare how known outbreak strains were classified by WGS relative to MLST and PFGE. Notably, the WGS analyses identified some strains that should have been included in prior outbreak investigations but were misclassified by PFGE, as well as some genetically unrelated strains with similar PFGE banding patterns. The application of hqSNP analysis on a subset of isolates also promoted the identification of clusters with higher discriminatory power. For these analyses, we sought to create cutoffs based on SNP differences to detect clusters of related isolates and exclude isolates with slightly more SNP differences. These efforts led to the identification of putative outbreak strains with missing epidemiological links. For example, the analysis of O157 strains from the ST-66-O1 outbreak identified one highly similar strain that was isolated one year prior (Figure 4). Similarly, two O157 strains isolated from distinct outbreaks, ST-66-02 and ST-66-O3, were nearly identical in the hqSNP analysis despite being isolated two years apart (Figure 5). Although the epidemiological linkages may have been lacking in some of these cases, the findings from both analyses highlight the possibility of a continuous source outbreak, which was not investigated at the time. Indeed, some food sources have a longer shelf life that could contribute to prolonged exposures, as was observed for the *Salmonella* serovar Tennessee outbreak in peanut butter [52]. Comparatively, the lack of cutoffs may result in the identification of smaller clusters that misclassify some isolates as outbreak-associated even without supporting epidemiological data. Establishing meaningful cutoffs will continue to change and vary by pathogen, though the goal of each analysis should be to determine which isolates and cases should be examined more comprehensively in outbreak investigations and source attribution studies.

As was shown in our prior study [17], MLST grouped the 509 STEC isolates into three clades, with Clade I comprising multiple subclades of strains with varying serogroups. Although MLST is beneficial to examine genetic diversity within the non-O157 STEC population, the discriminatory power is low for O157 strains [53,54,55]. For these non-O157 strains, the ST and serogroup designations are not always in agreement with placement in a MLST-based tree, and different MLST schemes are sometimes used. In our analysis, isolates from one serogroup were represented by multiple STs, while eleven different serogroups clustered together within the tree. These discrepancies could be indicative of recombination, which we described previously [17] but is not accounted for in either the MLST or cgSNP trees. The horizontal transfer of genes encoding the O-antigen could also result in serotype differences among strains of the same lineage [56,57]. Together, these results show that characterizing only the serogroup and ST cannot always differentiate strains to confirm that epidemiologically linked isolates are part of the same outbreak or cluster. A follow-up evaluation of cgSNPs is therefore recommended, which will promote strain differentiation and has been reliably used for both outbreak investigations and phylogenetic reconstructions in prior studies [34,58].

Because MLST loci are easy to extract from genomes and the subsequent analyses are feasible for use in a public health setting, MLST represents an efficient way to initially identify clusters of related strains that can be interrogated further. The identification of clustered isolates with few SNP differences may initially suggest that the strains are related; however, an epidemiological investigation and additional analyses are still required to confirm the link between the strains and identify potential outbreak sources. The inclusion of other data, such as the type of antibiotic resistance genes and virulence genes, can also be informative during an investigation, particularly for strains with supporting epidemiological data. In this analysis, most of the outbreak-associated strains had identical virulence gene alleles. The same is true for the placement of strains with strong epidemiological linkages within a MLST tree versus a cgSNP-based phylogeny. To this point, we observed no discrepancies in the phylogenetic relationships among STEC recovered from a small number of cattle living on the same farm over an eight-week period [38]. Since the 509 strains included in this study were highly diverse by MLST and mostly contributed to sporadic infections, it is probable that a complete cgSNP analysis could identify additional discrepancies and should be pursued in the future. Such analyses will also be important to enhance our understanding of the evolutionary history of STEC in this region.

Linking WGS data to existing PFGE data is also helpful to identify discrepancies; such comparative studies are important for refining the WGS analytical methods to be used in outbreak investigations in the future. Strains with similar PFGE patterns that group together in different parts of the cgSNP and hqSNP phylogenies can occur because of mutations in the genome that do not affect the *Xba*I restriction sites or drastically change the size of the fragments. Insertions and deletions of a few nucleotides are too minute to be accurately detected by gel electrophoresis [42]. At the same time, strains with distinct PFGE patterns that clustered together in the WGS analyses could occur due to changes at the restriction enzyme sites or methylation of the DNA [59]. Hence, the ability for PFGE to accurately identify strains that are similar is reliant on restriction enzyme sites remaining unmodified by genetic mutations. In this study, we often observed concordance between the MLST clusters and PFGE profiles, though WGS showed that some of the strains were not actually related. This discrepancy is likely due to the higher discriminatory power of WGS or to human error, particularly for PFGE. The two ST-106 isolates (PNUSAE001592 and PNUSAE001586), for example, had identical PFGE patterns but were distinct in the MLST, cgSNP, and hqSNP analyses, as well as the complete genome analysis that identified >25,000 SNP differences. Conversely, the hqSNP phylogeny of ST-119 isolates identified a cluster of isolates that differed by only 19–90 SNPs, even though all strains had distinct PFGE patterns. These isolates clustered together in the MLST tree and cgSNP phylogeny but exhibited slight differences in the hqSNP analysis, which are reflected by the PFGE patterns. Most importantly, WGS accurately differentiated the O5 ST-175 and O157 ST-66 outbreak strains, which clustered together in both SNP analyses and were identical by PFGE.

Collectively, our data further highlight the need for a transition to WGS to enhance outbreak surveillance activities and more accurately identify isolates that should be pursued in epidemiological investigations. Implementing WGS in public health laboratories will allow for more rapid characterization of foodborne pathogens and facilitate the extraction of genes encoding virulence factors, such as toxins and the O-antigen, as well as antibiotic resistance genes, to develop a preliminary assessment of virulence and susceptibilities. At the same time, the genetic relatedness of strains can be deduced to identify subsets of isolates for source tracking and downstream epidemiological investigations. Continued surveillance of STEC genomes in Michigan and elsewhere is needed as it will enhance our ability to rapidly monitor strain types in circulation and identify emergent types linked to clinical illness and more severe infections.

## Data Availability

The genomes analyzed in this study are available in the National Center for Biotechnology Information within BioProject PRJNA218110, which is available at: www.ncbi.nlm.nih.gov/bioproject/218110 (accessed on 1 September 2019). BioSample identification numbers pertaining to each strain, which is designated by the CDC PulseNet identification number, are listed in Table S2.

## Supplementary Materials

The following supporting information can be downloaded at: https://www.mdpi.com/article/10.3390/microorganisms11051298/s1, Figure S1: Flowchart of the bioinformatic tools used to classify 625 STEC or *Shigella* isolates submitted for WGS by the Michigan Department of Health and Human Services. Figure S2: Core genome (cg) SNP phylogeny of 137 STEC strains belonging to subclades D and E of Clade I in the multilocus sequence typing (MLST) tree. The inset shows pulsed-field gel electrophoresis (PFGE) patterns for the two isolates, PNUSAE001586 (O26:H11 ST-106) and PNUSAE001592 (NT:H19 ST-254), which were previously classified as part of the same outbreak. Both outbreak strains are denoted with stars and are represented in different clusters within the cgSNP phylogeny. Figure S3: cgSNP phylogeny of 186 STEC strains belonging to subclade A within Clade I in the MLST tree. All but two strains were classified as ST-119, while the stars highlight the placement of the two O103:H2 outbreak associated strains, PNUSAE004161 and PNUSAE004654, in the cgSNP phylogeny. Figure S4: cgSNP analysis of 98 STEC O157:H7 strains classified as ST-66. Sixteen strains from three outbreaks, denoted as ST-66-O1 (open star), ST-66-O2 (black star), and ST-66-O3 (open triangle) were included. Figure S5: cgSNP analysis of 17 STEC strains belonging to subclade G within Clade I in the MLST tree. Outbreak-associated strains belonging to serogroup O5:H9 are indicated with black stars. Table S1: Number of STEC serogroups detected in Michigan between 2015 and 2018 for WGS. Table S2: Accession numbers for STEC strains used in the study. Sequences were submitted to the NCBI as part of BioProject PRJNA218110 available at: www.ncbi.nlm.nih.gov/bioproject/218110 (accessed on 10 September 2019).
