# Supplementary material for: Whole-Genome Sequencing of Shiga Toxin-Producing Escherichia coli for Characterization and Outbreak Investigation
Source: Microorganisms. 2023 May 16;11(5):1298. doi: 10.3390/microorganisms11051298 (PMC10224053; doi:10.3390/microorganisms11051298)
Supplement: Supplementary file 1 [file microorganisms-11-01298-s001.zip › microorganisms-2356475-supplementary.pdf]

Blankenship et al. Supplemental Data

Figure S1. Flowchart of the bioinformatic tools used to classify 625 Shiga toxin-producing *Escherichia coli* (STEC) isolates or *Shigella* isolates submitted for whole-genome sequencing (WGS) by the Michigan Department of Health and Human Services.

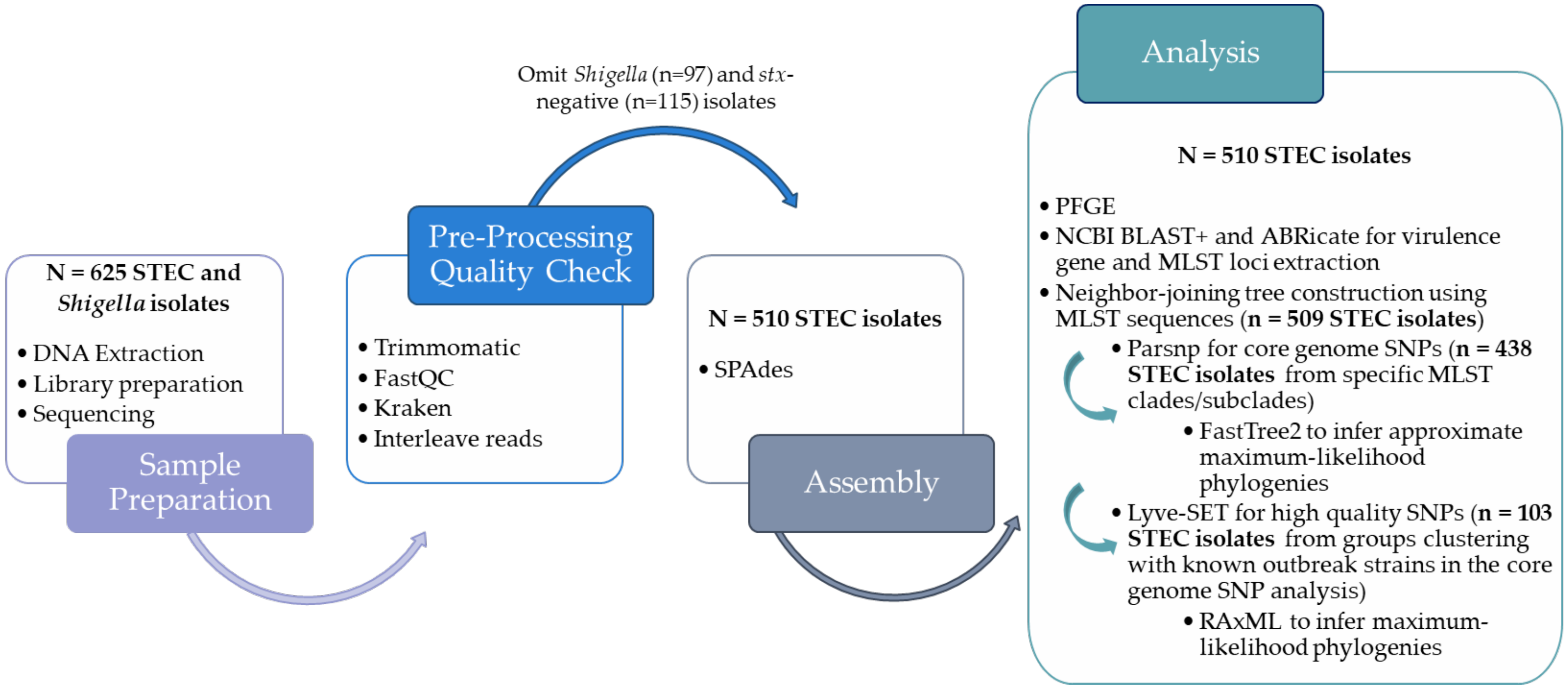

Figure S2. Core genome (cg) SNP phylogeny of 137 STEC strains belonging to subclades D and E of Clade I in the multilocus sequence typing (MLST) tree. The inset shows pulsed-field gel electrophoresis (PFGE) patterns for the two isolates, PNUSAE001586 (O26:H11 ST-106) and PNUSAE001592 (NT:H19 ST-254), which were previously classified as part of the same outbreak. Both outbreak strains are denoted with stars and are represented in different clusters within the cgSNP phylogeny.

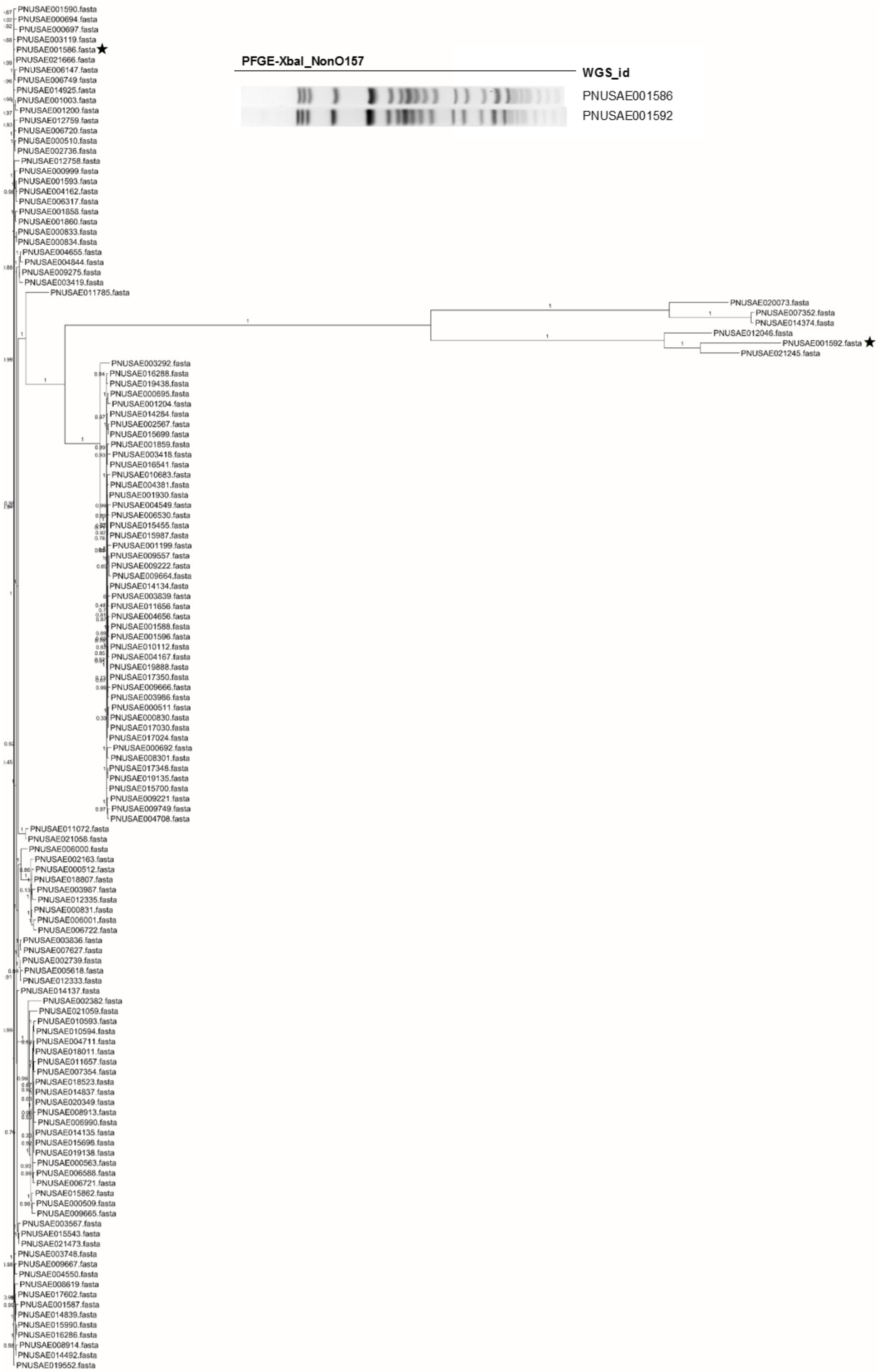

Figure S3. cgSNP phylogeny of 186 STEC strains belonging to subclade A within Clade I in the MLST tree. All but two strains were classified as ST-119, while the stars highlight the placement of the two O103:H2 outbreak associated strains, PNUSAE004161 and PNUSAE004654, in the cgSNP phylogeny.

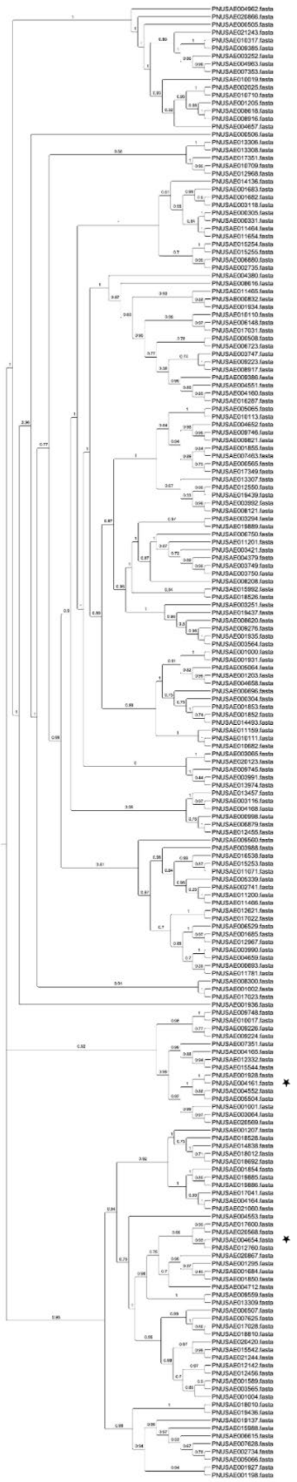

Figure S4. cgSNP analysis of 98 STEC O157:H7 strains classified as ST-66. Sixteen strains from three outbreaks, denoted as ST-66-O1 (open star), ST-66-O2 (black star), and ST-66-O3 (open triangle), were included in the analysis.

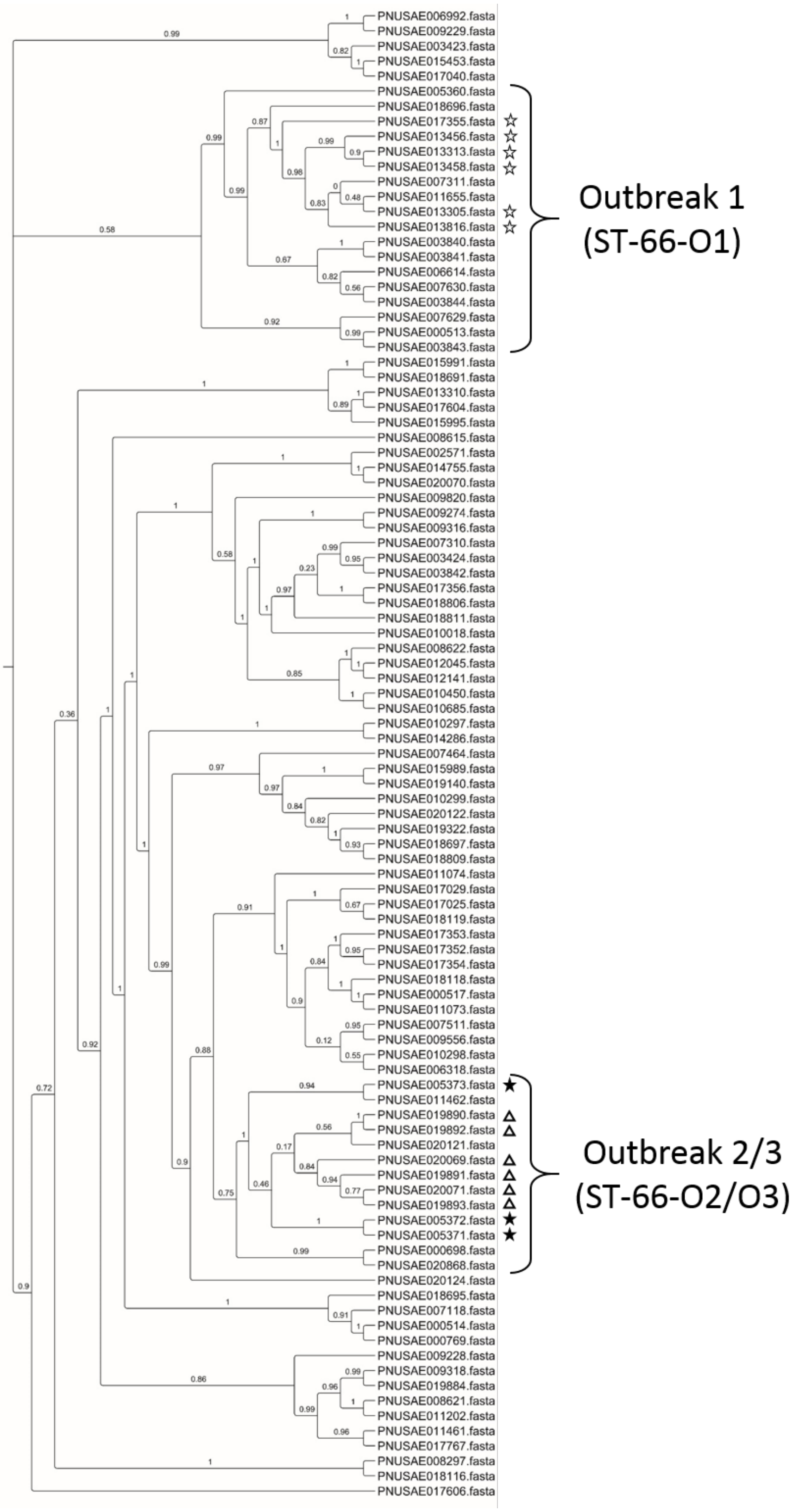

Figure S5. cgSNP analysis of 17 STEC strains belonging to subclade G within Clade I in the MLST tree. Outbreak-associated strains belonging to serogroup O5:H9 are indicated with black stars.

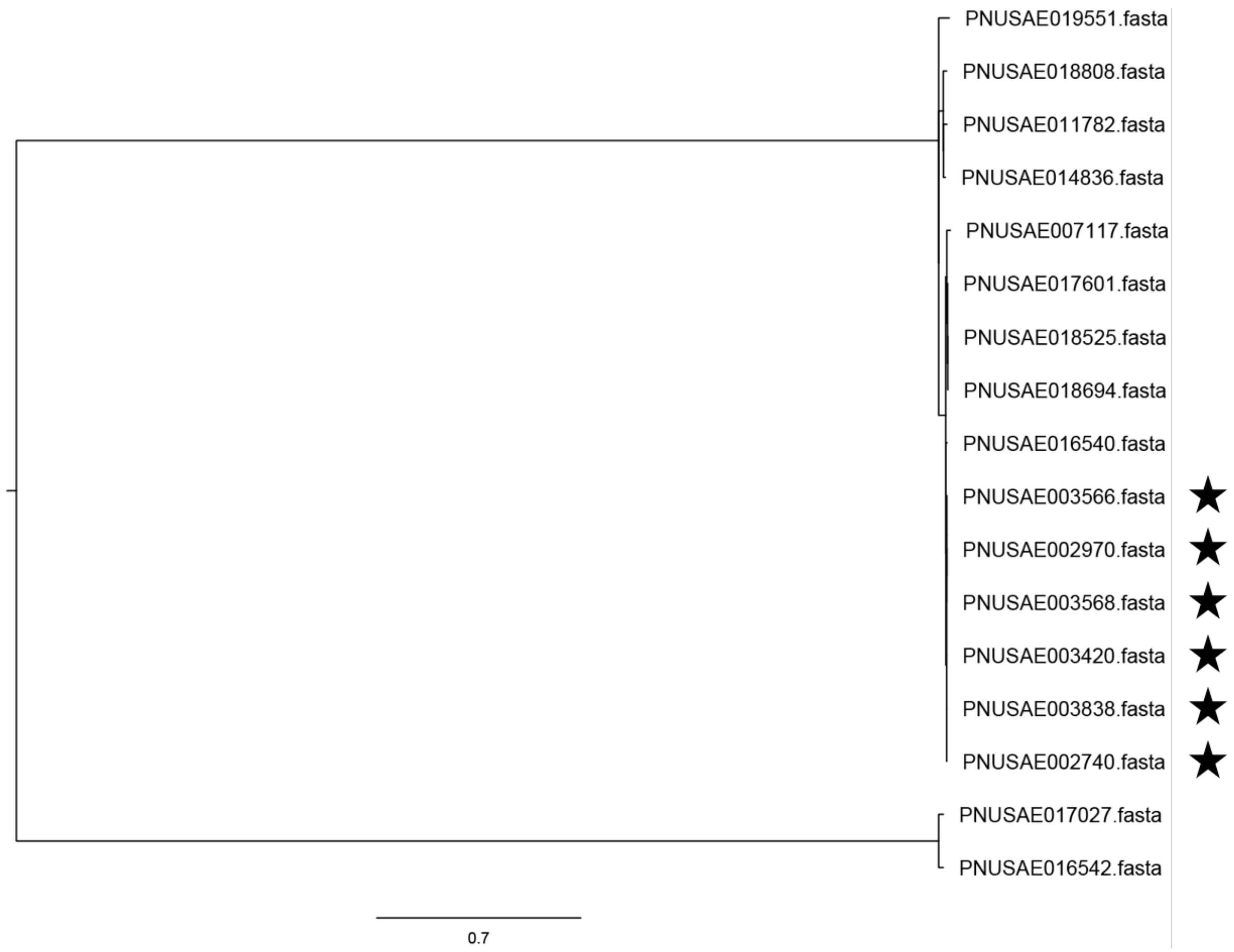

Table S1: Number of STEC serogroups detected in Michigan between 2015 and 2018 for WGS.

	Year				
**Serogroup**	**2015**	**2016**	**2017**	**2018**	**Total**
**O5**	0	6	2	7	15
**O17**	0	3	0	3	6
**O28ac**	0	1	0	1	2
**O55**	0	2	0	0	2
**O69**	1	0	1	1	3
**O71**	1	1	8	7	17
**O73 or O17/O77**	0	0	1	1	2
**O8**	0	0	0	3	3
**O80**	0	0	0	1	1
**O84**	0	1	2	1	4
**O85**	0	0	1	0	1
**O98**	0	0	0	1	1
**O109**	0	1	0	0	1
**O113**	0	0	1	0	1
**O115**	0	1	0	0	1
**O117**	0	1	0	0	1
**O118**	0	0	0	1	1
**O123**	4	3	3	3	13
**O130**	0	1	0	0	1
**O151**	2	3	3	2	10
**O153/O178**	0	0	0	1	1
**O156**	0	0	0	2	2
**O165**	0	0	1	1	2
**O166**	0	1	0	0	1
**O172**	0	0	0	1	1
**O177**	0	0	0	3	3
**O183**	0	0	1	2	3
**O26**	14	12	10	15	51
**O45**	13	16	21	11	61
**O103**	21	23	26	41	111
**O111**	10	10	11	16	47
**O121**	8	13	6	1	28
**O145**	0	2	0	4	6
**O157**	5	11	33	49	98
**NT**	5	0	1	3	9
**Totals**	84	112	132	182	510

Table S2. Accession numbers for STEC strains used in the study. Sequences were submitted to the National Center for Biotechnology Information (NCBI) as part of BioProject PRJNA218110 available at: www.ncbi.nlm.nih.gov/bioproject/218110 (accessed on 1 September 2019).

NCBI BioSample	CDC PulseNet Strain Name
SAMN03382866	PNUSAE000304
SAMN03382867	PNUSAE000305
SAMN03382869	PNUSAE000331
SAMN03492160	PNUSAE000505
SAMN03492161	PNUSAE000506
SAMN03492162	PNUSAE000507
SAMN03492163	PNUSAE000508
SAMN03492164	PNUSAE000509
SAMN03492165	PNUSAE000510
SAMN03492166	PNUSAE000511
SAMN03492167	PNUSAE000512
SAMN03610070	PNUSAE000563
SAMN03610071	PNUSAE000564
SAMN03610072	PNUSAE000565
SAMN03610079	PNUSAE000513
SAMN03610080	PNUSAE000517
SAMN03763865	PNUSAE000692
SAMN03763866	PNUSAE000693
SAMN03763867	PNUSAE000694
SAMN03763868	PNUSAE000695
SAMN03763869	PNUSAE000697
SAMN03763870	PNUSAE000696
SAMN03763871	PNUSAE000698
SAMN03795200	PNUSAE000514
SAMN03795203	PNUSAE000769
SAMN03795204	PNUSAE000830
SAMN03795205	PNUSAE000831
SAMN03795206	PNUSAE000832
SAMN03795207	PNUSAE000833
SAMN03795208	PNUSAE000834
SAMN03857010	PNUSAE000998
SAMN03857011	PNUSAE000999
SAMN03857012	PNUSAE001000
SAMN03857013	PNUSAE001001
SAMN03857014	PNUSAE001002
SAMN03857015	PNUSAE001003
SAMN03857016	PNUSAE001004

NCBI BioSample	CDC PulseNet Strain Name
SAMN03981626	PNUSAE001203
SAMN03981627	PNUSAE001198
SAMN03981628	PNUSAE001199
SAMN03981629	PNUSAE001200
SAMN03981630	PNUSAE001202
SAMN03981631	PNUSAE001204
SAMN03981632	PNUSAE001206
SAMN04010054	PNUSAE001207
SAMN04010055	PNUSAE001208
SAMN04010056	PNUSAE001295
SAMN04191663	PNUSAE001586
SAMN04191664	PNUSAE001587
SAMN04191665	PNUSAE001588
SAMN04191666	PNUSAE001589
SAMN04191667	PNUSAE001590
SAMN04191668	PNUSAE001592
SAMN04191669	PNUSAE001593
SAMN04191670	PNUSAE001594
SAMN04191671	PNUSAE001595
SAMN04191672	PNUSAE001596
SAMN04230435	PNUSAE001591
SAMN04230436	PNUSAE001682
SAMN04230437	PNUSAE001683
SAMN04230438	PNUSAE001684
SAMN04230439	PNUSAE001685
SAMN04335022	PNUSAE001850
SAMN04335024	PNUSAE001852
SAMN04335025	PNUSAE001853
SAMN04335026	PNUSAE001854
SAMN04335027	PNUSAE001858
SAMN04335028	PNUSAE001859
SAMN04335029	PNUSAE001860
SAMN04335031	PNUSAE001855
SAMN04351182	PNUSAE001857
SAMN04351183	PNUSAE001934
SAMN04351184	PNUSAE001935
SAMN04351185	PNUSAE001936
SAMN04351186	PNUSAE001928
SAMN04351187	PNUSAE001929
SAMN04351188	PNUSAE001930

NCBI BioSample	CDC PulseNet Strain Name
SAMN04351189	PNUSAE001927
SAMN04351190	PNUSAE001933
SAMN04351191	PNUSAE001931
SAMN04351192	PNUSAE001932
SAMN04445972	PNUSAE002162
SAMN04445980	PNUSAE002163
SAMN04518090	PNUSAE002380
SAMN04518092	PNUSAE002382
SAMN04518098	PNUSAE002025
SAMN04568477	PNUSAE002567
SAMN04568478	PNUSAE002568
SAMN04568479	PNUSAE002569
SAMN04568480	PNUSAE002570
SAMN04568481	PNUSAE002571
SAMN04632362	PNUSAE002734
SAMN04632363	PNUSAE002735
SAMN04632364	PNUSAE002736
SAMN04632365	PNUSAE002737
SAMN04632366	PNUSAE002738
SAMN04632367	PNUSAE002739
SAMN04632368	PNUSAE002740
SAMN04632369	PNUSAE002741
SAMN04931569	PNUSAE002970
SAMN04962256	PNUSAE003064
SAMN04962257	PNUSAE003065
SAMN05163612	PNUSAE003117
SAMN05163613	PNUSAE003118
SAMN05163614	PNUSAE003119
SAMN05163615	PNUSAE003115
SAMN05201340	PNUSAE003250
SAMN05201341	PNUSAE003251
SAMN05201342	PNUSAE003252
SAMN05201343	PNUSAE003253
SAMN05201344	PNUSAE003254
SAMN05225544	PNUSAE003292
SAMN05225545	PNUSAE003293
SAMN05225546	PNUSAE003294
SAMN05292333	PNUSAE003424
SAMN05292334	PNUSAE003417
SAMN05292335	PNUSAE003419
SAMN05292336	PNUSAE003423

NCBI BioSample	CDC PulseNet Strain Name
SAMN05292337	PNUSAE003420
SAMN05292338	PNUSAE003421
SAMN05372176	PNUSAE003565
SAMN05372177	PNUSAE003566
SAMN05372178	PNUSAE003567
SAMN05372179	PNUSAE003418
SAMN05432126	PNUSAE003747
SAMN05432127	PNUSAE003748
SAMN05432128	PNUSAE003749
SAMN05432138	PNUSAE003564
SAMN05432139	PNUSAE003568
SAMN05505246	PNUSAE003840
SAMN05505247	PNUSAE003841
SAMN05505248	PNUSAE003842
SAMN05505249	PNUSAE003843
SAMN05505250	PNUSAE003844
SAMN05505251	PNUSAE003835
SAMN05505252	PNUSAE003836
SAMN05505253	PNUSAE003837
SAMN05505254	PNUSAE003839
SAMN05505255	PNUSAE003750
SAMN05560426	PNUSAE003986
SAMN05560427	PNUSAE003838
SAMN05560428	PNUSAE003988
SAMN05560429	PNUSAE003989
SAMN05560430	PNUSAE003990
SAMN05560431	PNUSAE003992
SAMN05660005	PNUSAE004162
SAMN05660006	PNUSAE004165
SAMN05660007	PNUSAE004166
SAMN05660008	PNUSAE004168
SAMN05660013	PNUSAE004160
SAMN05751972	PNUSAE004381
SAMN05751982	PNUSAE004379
SAMN05805028	PNUSAE004549
SAMN05805029	PNUSAE004380
SAMN05805030	PNUSAE003991
SAMN05805031	PNUSAE004167
SAMN05805032	PNUSAE004550
SAMN05805033	PNUSAE004551

NCBI BioSample	CDC PulseNet Strain Name
SAMN05805034	PNUSAE004552
SAMN05805035	PNUSAE004553
SAMN05859320	PNUSAE004652
SAMN05859321	PNUSAE004653
SAMN05859322	PNUSAE004654
SAMN05859323	PNUSAE004655
SAMN05859324	PNUSAE004656
SAMN05859325	PNUSAE004657
SAMN05859326	PNUSAE004659
SAMN05859327	PNUSAE004161
SAMN05859328	PNUSAE004163
SAMN05859329	PNUSAE004164
SAMN05879162	PNUSAE004708
SAMN05879163	PNUSAE004709
SAMN05879164	PNUSAE004710
SAMN05879165	PNUSAE004711
SAMN05879166	PNUSAE004712
SAMN05914957	PNUSAE004658
SAMN05914958	PNUSAE004787
SAMN05933680	PNUSAE004844
SAMN05978632	PNUSAE004962
SAMN05978633	PNUSAE004963
SAMN05978634	PNUSAE004964
SAMN06020427	PNUSAE005066
SAMN06020431	PNUSAE005064
SAMN06111355	PNUSAE005338
SAMN06111356	PNUSAE005339
SAMN06145158	PNUSAE005065
SAMN06145162	PNUSAE005371
SAMN06145163	PNUSAE005372
SAMN06145164	PNUSAE005373
SAMN06145165	PNUSAE005360
SAMN06174471	PNUSAE005504
SAMN06212806	PNUSAE005617
SAMN06212807	PNUSAE005618
SAMN06324315	PNUSAE006000
SAMN06449135	PNUSAE006001
SAMN06449141	PNUSAE006147
SAMN06449142	PNUSAE006148
SAMN06464031	PNUSAE001205
SAMN06606893	PNUSAE006317

NCBI BioSample	CDC PulseNet Strain Name
SAMN06677638	PNUSAE006529
SAMN06677639	PNUSAE006588
SAMN06677640	PNUSAE006530
SAMN06701394	PNUSAE006615
SAMN06701395	PNUSAE006614
SAMN06704733	PNUSAE003987
SAMN06759798	PNUSAE006720
SAMN06759799	PNUSAE006721
SAMN06759800	PNUSAE006722
SAMN06806028	PNUSAE006723
SAMN06843059	PNUSAE006749
SAMN06843060	PNUSAE006750
SAMN06855417	PNUSAE006879
SAMN06855428	PNUSAE003116
SAMN06898047	PNUSAE006880
SAMN06911692	PNUSAE006318
SAMN07139316	PNUSAE007118
SAMN07143102	PNUSAE006989
SAMN07143111	PNUSAE006992
SAMN07172902	PNUSAE006991
SAMN07172904	PNUSAE006990
SAMN07173337	PNUSAE007117
SAMN07193415	PNUSAE007310
SAMN07193416	PNUSAE007351
SAMN07193417	PNUSAE007353
SAMN07193418	PNUSAE007354
SAMN07216945	PNUSAE007311
SAMN07216946	PNUSAE007352
SAMN07216955	PNUSAE007464
SAMN07216956	PNUSAE007463
SAMN07246882	PNUSAE007511
SAMN07303548	PNUSAE007625
SAMN07303549	PNUSAE007626
SAMN07303550	PNUSAE008121
SAMN07303551	PNUSAE007629
SAMN07303552	PNUSAE007630
SAMN07303553	PNUSAE007628
SAMN07346315	PNUSAE007627
SAMN07349421	PNUSAE008297
SAMN07349875	PNUSAE008299

NCBI BioSample	CDC PulseNet Strain Name
SAMN07349876	PNUSAE008300
SAMN07349885	PNUSAE008207
SAMN07362151	PNUSAE008298
SAMN07373014	PNUSAE008301
SAMN07450893	PNUSAE008614
SAMN07450894	PNUSAE008615
SAMN07450895	PNUSAE008616
SAMN07450896	PNUSAE008621
SAMN07450909	PNUSAE008622
SAMN07450910	PNUSAE008617
SAMN07450911	PNUSAE008618
SAMN07450912	PNUSAE008619
SAMN07450913	PNUSAE008620
SAMN07508916	PNUSAE008917
SAMN07519426	PNUSAE008916
SAMN07523017	PNUSAE009228
SAMN07523018	PNUSAE009229
SAMN07523019	PNUSAE009221
SAMN07523020	PNUSAE009222
SAMN07523021	PNUSAE009223
SAMN07523022	PNUSAE009224
SAMN07523024	PNUSAE009226
SAMN07554541	PNUSAE008913
SAMN07554542	PNUSAE008914
SAMN07561509	PNUSAE009275
SAMN07561510	PNUSAE009274
SAMN07561511	PNUSAE009276
SAMN07567275	PNUSAE009318
SAMN07624152	PNUSAE009557
SAMN07624153	PNUSAE009558
SAMN07624154	PNUSAE009556
SAMN07624155	PNUSAE009560
SAMN07624163	PNUSAE009385
SAMN07624164	PNUSAE009386
SAMN07629708	PNUSAE009664
SAMN07629709	PNUSAE009665
SAMN07629710	PNUSAE009666
SAMN07647586	PNUSAE009559
SAMN07647587	PNUSAE009667
SAMN07663082	PNUSAE009747
SAMN07663083	PNUSAE009820

NCBI BioSample	CDC PulseNet Strain Name
SAMN07663084	PNUSAE009821
SAMN07721579	PNUSAE009745
SAMN07721580	PNUSAE009746
SAMN07750440	PNUSAE010017
SAMN07750441	PNUSAE010018
SAMN07750442	PNUSAE010019
SAMN07750443	PNUSAE010110
SAMN07750449	PNUSAE009749
SAMN07750450	PNUSAE010111
SAMN07750451	PNUSAE010112
SAMN07750452	PNUSAE010113
SAMN07788950	PNUSAE009748
SAMN07816711	PNUSAE010298
SAMN07816712	PNUSAE010299
SAMN07816713	PNUSAE010317
SAMN07816714	PNUSAE010297
SAMN07842331	PNUSAE010593
SAMN07842332	PNUSAE010594
SAMN07967330	PNUSAE008208
SAMN07967331	PNUSAE010450
SAMN07967334	PNUSAE010685
SAMN07967335	PNUSAE010682
SAMN07967337	PNUSAE010683
SAMN07967338	PNUSAE010709
SAMN08027469	PNUSAE010684
SAMN08100391	PNUSAE011071
SAMN08100392	PNUSAE011072
SAMN08100393	PNUSAE011074
SAMN08128621	PNUSAE011159
SAMN08128622	PNUSAE011200
SAMN08128623	PNUSAE011202
SAMN08136953	PNUSAE011073
SAMN08202707	PNUSAE010710
SAMN08202708	PNUSAE011201
SAMN08202709	PNUSAE011463
SAMN08202710	PNUSAE011464
SAMN08202711	PNUSAE011462
SAMN08225344	PNUSAE011461
SAMN08225345	PNUSAE011465
SAMN08225346	PNUSAE011466

NCBI BioSample	CDC PulseNet Strain Name
SAMN08299193	PNUSAE011657
SAMN08299194	PNUSAE011656
SAMN08299195	PNUSAE011655
SAMN08299196	PNUSAE011654
SAMN08359589	PNUSAE011785
SAMN08359593	PNUSAE011781
SAMN08359594	PNUSAE011782
SAMN08359595	PNUSAE011783
SAMN08451861	PNUSAE012046
SAMN08451862	PNUSAE012045
SAMN08499982	PNUSAE012141
SAMN08595226	PNUSAE012332
SAMN08595241	PNUSAE012335
SAMN08595242	PNUSAE012333
SAMN08610765	PNUSAE012331
SAMN08646836	PNUSAE012456
SAMN08646837	PNUSAE012455
SAMN08743511	PNUSAE012621
SAMN08743512	PNUSAE012550
SAMN08743514	PNUSAE012142
SAMN08796833	PNUSAE012757
SAMN08799703	PNUSAE012760
SAMN08799704	PNUSAE012759
SAMN08799706	PNUSAE012758
SAMN08868516	PNUSAE012968
SAMN08868517	PNUSAE012967
SAMN08964481	PNUSAE013310
SAMN08964482	PNUSAE013309
SAMN08964483	PNUSAE013306
SAMN08964484	PNUSAE013305
SAMN08971255	PNUSAE013308
SAMN08971842	PNUSAE013313
SAMN08971845	PNUSAE013312
SAMN09012044	PNUSAE013458
SAMN09012045	PNUSAE013457
SAMN09012046	PNUSAE013456
SAMN09092835	PNUSAE013816
SAMN09092836	PNUSAE013307
SAMN09229873	PNUSAE013974
SAMN09238033	PNUSAE014134
SAMN09238051	PNUSAE014137

NCBI BioSample	CDC PulseNet Strain Name
SAMN09238054	PNUSAE014136
SAMN09281082	PNUSAE014286
SAMN09281083	PNUSAE014285
SAMN09281084	PNUSAE014284
SAMN09289793	PNUSAE014374
SAMN09289794	PNUSAE014135
SAMN09289795	PNUSAE012334
SAMN09375717	PNUSAE014493
SAMN09405439	PNUSAE014755
SAMN09474595	PNUSAE014492
SAMN09489318	PNUSAE014925
SAMN09489319	PNUSAE014839
SAMN09489320	PNUSAE014837
SAMN09489321	PNUSAE014836
SAMN09489762	PNUSAE014838
SAMN09489766	PNUSAE014835
SAMN09533790	2017C-4625C1
SAMN09623419	PNUSAE015455
SAMN09623420	PNUSAE015453
SAMN09623421	PNUSAE015255
SAMN09623422	PNUSAE015254
SAMN09623423	PNUSAE015253
SAMN09637202	PNUSAE015544
SAMN09637203	PNUSAE015542
SAMN09637221	PNUSAE015543
SAMN09691739	PNUSAE015862
SAMN09697972	PNUSAE015700
SAMN09697975	PNUSAE015699
SAMN09697977	PNUSAE015698
SAMN09740356	PNUSAE016284
SAMN09753001	PNUSAE015995
SAMN09753004	PNUSAE015991
SAMN09753005	PNUSAE015989
SAMN09753006	PNUSAE015988
SAMN09753007	PNUSAE015987
SAMN09753136	PNUSAE015990
SAMN09763529	PNUSAE016288
SAMN09763530	PNUSAE016287
SAMN09763531	PNUSAE016286
SAMN09813306	PNUSAE017024

NCBI BioSample	CDC PulseNet Strain Name
SAMN09813308	PNUSAE017023
SAMN09813309	PNUSAE017022
SAMN09813318	PNUSAE017041
SAMN09813319	PNUSAE017040
SAMN09813320	PNUSAE017031
SAMN09813322	PNUSAE017030
SAMN09813323	PNUSAE017029
SAMN09813325	PNUSAE017028
SAMN09813326	PNUSAE017027
SAMN09813327	PNUSAE017026
SAMN09813329	PNUSAE017025
SAMN09878826	PNUSAE017354
SAMN09878827	PNUSAE017353
SAMN09878828	PNUSAE017352
SAMN09878829	PNUSAE017351
SAMN09878830	PNUSAE017350
SAMN09878831	PNUSAE017349
SAMN09878832	PNUSAE017348
SAMN09878841	PNUSAE017356
SAMN09878842	PNUSAE017355
SAMN09910495	PNUSAE017601
SAMN09910539	PNUSAE017602
SAMN09934159	PNUSAE016542
SAMN09934160	PNUSAE016541
SAMN09934161	PNUSAE016540
SAMN09934163	PNUSAE016538
SAMN09979469	PNUSAE017604
SAMN09979470	PNUSAE017600
SAMN09979483	PNUSAE017606
SAMN09979484	PNUSAE017605
SAMN09986376	PNUSAE017603
SAMN09986383	PNUSAE017767
SAMN10038191	PNUSAE018116
SAMN10038192	PNUSAE018118
SAMN10112217	PNUSAE018528
SAMN10112218	PNUSAE018527
SAMN10112219	PNUSAE018526
SAMN10112220	PNUSAE018525
SAMN10112221	PNUSAE018119
SAMN10112225	PNUSAE018523
SAMN10139577	PNUSAE018806

NCBI BioSample	CDC PulseNet Strain Name
SAMN10139578	PNUSAE018694
SAMN10139579	PNUSAE018696
SAMN10139580	PNUSAE018693
SAMN10139581	PNUSAE018691
SAMN10139593	PNUSAE018811
SAMN10139594	PNUSAE018697
SAMN10139595	PNUSAE018810
SAMN10139596	PNUSAE018809
SAMN10139597	PNUSAE018807
SAMN10139599	PNUSAE018695
SAMN10227572	PNUSAE019140
SAMN10227573	PNUSAE019139
SAMN10227574	PNUSAE019138
SAMN10227575	PNUSAE019137
SAMN10227576	PNUSAE019135
SAMN10231735	PNUSAE019322
SAMN10267413	PNUSAE019438
SAMN10267414	PNUSAE019437
SAMN10267415	PNUSAE019436
SAMN10267416	PNUSAE018808
SAMN10267417	PNUSAE015992
SAMN10279432	PNUSAE019554
SAMN10279433	PNUSAE019553
SAMN10279434	PNUSAE019552
SAMN10279803	PNUSAE019551
SAMN10352909	PNUSAE019439
SAMN10391782	PNUSAE019887
SAMN10391783	PNUSAE019884
SAMN10391785	PNUSAE019892
SAMN10391786	PNUSAE019890
SAMN10391787	PNUSAE019889
SAMN10391788	PNUSAE019888
SAMN10391795	PNUSAE020073
SAMN10391796	PNUSAE020072
SAMN10391797	PNUSAE020071
SAMN10391798	PNUSAE020070
SAMN10391799	PNUSAE020069
SAMN10432149	PNUSAE020124
SAMN10432150	PNUSAE020122
SAMN10432151	PNUSAE020121

NCBI BioSample	CDC PulseNet Strain Name
SAMN10432152	PNUSAE019891
SAMN10432153	PNUSAE020120
SAMN10432154	PNUSAE019886
SAMN10432284	PNUSAE020123
SAMN10449172	PNUSAE020349
SAMN10503925	PNUSAE020569
SAMN10503926	PNUSAE020568
SAMN10619093	PNUSAE020420
SAMN10619107	PNUSAE020868
SAMN10619108	PNUSAE020867
SAMN10619110	PNUSAE020866
SAMN10661712	PNUSAE021245
SAMN10661713	PNUSAE021244
SAMN10661714	PNUSAE021060
SAMN10661715	PNUSAE021058
SAMN10661716	PNUSAE021059
SAMN10700124	PNUSAE018009
SAMN10700163	PNUSAE021358
SAMN10700164	PNUSAE018012
SAMN10700165	PNUSAE018011
SAMN10700166	PNUSAE018010
SAMN10774441	PNUSAE021243
SAMN10774615	PNUSAE019893
SAMN10774616	PNUSAE021666
SAMN10774617	PNUSAE021473
SAMN10774618	PNUSAE019885

Note: Sequences belonging to strains PNUSAE009316 and PNUSAE018692 are missing from the NCBI database
